# Visualization of Radial Peripapillary Capillaries Using Optical Coherence Tomography Angiography: The Effect of Image Averaging

**DOI:** 10.1371/journal.pone.0169385

**Published:** 2017-01-09

**Authors:** Shelley Mo, Erika Phillips, Brian D. Krawitz, Reena Garg, Sarwat Salim, Lawrence S. Geyman, Eleni Efstathiadis, Joseph Carroll, Richard B. Rosen, Toco Y. P. Chui

**Affiliations:** 1 Icahn School of Medicine at Mount Sinai, New York, New York, United States of America; 2 Department of Ophthalmology, New York Eye and Ear Infirmary of Mount Sinai, New York, New York, United States of America; 3 Department of Ophthalmology, Medical College of Wisconsin, Milwaukee, Wisconsin, United States of America; 4 William E. Macaulay Honors College, New York, New York, United States of America; 5 Department of Biophysics, Medical College of Wisconsin, Milwaukee, Wisconsin, United States of America; 6 Department of Cell Biology, Neurobiology & Anatomy, Medical College of Wisconsin, Milwaukee, Wisconsin, United States of America; Simon Fraser University, CANADA

## Abstract

**Objectives:**

To assess the effect of image registration and averaging on the visualization and quantification of the radial peripapillary capillary (RPC) network on optical coherence tomography angiography (OCTA).

**Methods:**

Twenty-two healthy controls were imaged with a commercial OCTA system (AngioVue, Optovue, Inc.). Ten 10x10° scans of the optic disc were obtained, and the most superficial layer (50-μm slab extending from the inner limiting membrane) was extracted for analysis. Rigid registration was achieved using ImageJ, and averaging of each 2 to 10 frames was performed in five ~2x2° regions of interest (ROI) located 1° from the optic disc margin. The ROI were automatically skeletonized. Signal-to-noise ratio (SNR), number of endpoints and mean capillary length from the skeleton, capillary density, and mean intercapillary distance (ICD) were measured for the reference and each averaged ROI. Repeated measures analysis of variance was used to assess statistical significance. Three patients with primary open angle glaucoma were also imaged to compare RPC density to controls.

**Results:**

Qualitatively, vessels appeared smoother and closer to histologic descriptions with increasing number of averaged frames. Quantitatively, number of endpoints decreased by 51%, and SNR, mean capillary length, capillary density, and ICD increased by 44%, 91%, 11%, and 4.5% from single frame to 10-frame averaged, respectively. The 10-frame averaged images from the glaucomatous eyes revealed decreased density correlating to visual field defects and retinal nerve fiber layer thinning.

**Conclusions:**

OCTA image registration and averaging is a viable and accessible method to enhance the visualization of RPCs, with significant improvements in image quality and RPC quantitative parameters. With this technique, we will be able to non-invasively and reliably study RPC involvement in diseases such as glaucoma.

## Introduction

The radial peripapillary capillaries (RPCs) make up a distinctive vascular network within the retinal nerve fiber layer (RNFL) around the optic disc. Their presence appears to be intimately tied to the highly metabolically active retinal ganglion cell axons that compose the RNFL [1–5]. Because of their parallel structure and paucity of anastomoses, RPCs are thought to be especially vulnerable to retinal pathologies such as glaucoma and retinal vascular occlusion [6, 7]. Though correlations between RNFL thickness and RPC volume have been found in previous studies, this neurovascular relationship remains poorly understood in disease. Studying this relationship *in vivo* has remained challenging due to the limitations of conventional clinical imaging tools, such as intravenous fluorescein angiography (IV FA). Adaptive optics scanning light ophthalmoscopy (AOSLO) equipped with fluorescein angiography or a nonconfocal detection scheme has had the most success with imaging RPCs *in vivo* [8–11]. However, the technique can be time-consuming and as such is not yet clinically optimized. The need for a clinically accessible and reliable method of visualizing this microvascular network is critical to help advance our understanding of ocular pathology and identify novel biomarkers for diagnosing these diseases.

Optical coherence tomography angiography (OCTA) is a novel technology that allows for non-invasive microvascular imaging through the use of motion contrast image processing techniques [12–20]. It has been validated against both histology and other *in vivo* vascular imaging including IV FA and AOSLO [21–26]. Several groups have proposed methods to quantify these angiographic images, including vessel area density [27–30], vessel skeleton density [27, 31], intercapillary distance [26], and non-perfused area [29].

However, limitations introduced by the optics of the eye, the optics of the imaging system, and eye movement during image acquisition challenge our ability to image small-caliber, dense capillary beds such as the RPCs [32]. One method to compensate for these issues may be through *en face* image averaging, echoing the technique used to improve OCT image quality [33–36]. Unlike previous applications of registration and averaging in OCTA which were largely focused on correcting for eye motion artifacts [34, 37–39], our proposed method would require that a patient undergo multiple scans in a single session for offline image registration and averaging. While scan times are short (6–8 seconds per OCTA) and the light exposure is limited, subjecting patients to multiple scans can be difficult and tiring. In order to obtain more reliable quantification of RPCs while maximizing patient comfort and minimizing duration of imaging sessions, we used image registration and averaging to enhance the quality of OCTA images and used quantitative methods to determine the optimal number of images needed for vascular analysis.

## Materials and Methods

### Subjects

This study was conducted in accordance with the tenets of the Declaration of Helsinki and was approved by the Institutional Review Boards at both the New York Eye and Ear Infirmary of Mount Sinai and the Medical College of Wisconsin. Written informed consent was obtained from all study participants. Twenty-two subjects with no history of ocular disease were recruited for this study. In addition, 3 patients with primary open angle glaucoma with defects on Humphrey visual fields (SITA Standard 24–2) were recruited. Diagnoses were determined through chart review. Inclusion criteria were as follows: normal anterior segment, clear natural lens, clear media, best corrected visual acuity of 20/80 or better in the eye imaged, and excellent fixation. Exclusion criteria included nuclear sclerosis greater than 2+ and presence of any systemic disease with ocular manifestations, such as diabetes. For subjects with pupils smaller than 5 mm in dim lighting, one drop of 2.5% phenylephrine hydrochloride ophthalmic solution (Paragon BioTeck, Inc., Portland, OR) and two drops of 1% tropicamide ophthalmic solution (Akorn, Inc., Lake Forest, IL) were administered to the eye imaged. In order to correct for individual retinal magnification, axial lengths were obtained using an IOL Master (Carl Zeiss Meditec, Inc., Dublin, CA) [40].

### OCTA and OCT image acquisition

All subjects were imaged using a commercial spectral domain OCT (RTVue XR Avanti, Optovue, Inc., Fremont, CA) with a scan rate of 70,000 A-scans/second, scan beam wavelength of 840 ± 10 nm, and bandwidth of 45 nm. The maximum exposure power at the pupil is 750 μW. OCTA images were generated using a standard commercial software (AngioVue version 2015.100.0.35, Optovue, Inc., Fremont, CA), which employs the split-spectrum amplitude decorrelation angiography algorithm [12]. Ten 10x10° (304x304 pixels) scans were obtained of the optic disc. The scanning raster was positioned to place the nasal disc margin adjacent to the edge of the image in order to expose more retinal area temporal to the disc. There were 304 A-scans per B-scan and 608 total B-scans per volumetric raster scan with 2 consecutive B-scans taken at each location. Each OCTA was composed of two volumetric raster scans (X-fast and Y-fast), each requiring 3–4 seconds. Subjects were encouraged to take breaks every 2–3 OCTAs or as needed. Circular OCT scans, 12° in diameter centered at the optic nerve head (Spectralis HRA+OCT, Heidelberg Engineering, Inc., Heidelberg, Germany), were obtained in order to measure peripapillary RNFL thickness in the three glaucomatous eyes. Central 24–2 Swedish Interactive Thresholding Algorithm (SITA) Standard Humphrey Visual Fields completed within 6 months of OCTA imaging were obtained from the three patients’ medical records.

### Image registration

**Fig 1:**
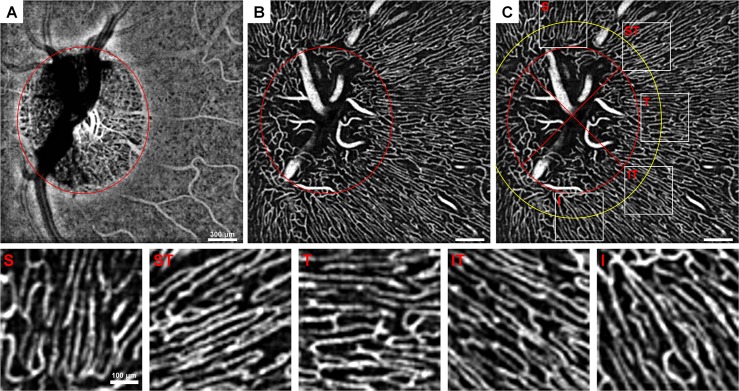
Peripapillary ROI selection demonstrated on 10-frame averaged OCTAs. (A) Choroidal OCTA and (B) superficial OCTA layers displaying the optic disc margin approximation (red ellipse). (C) Superficial layer with optic disc margin (red ellipse), 1° away from the optic disc margin (yellow ellipse), demarcation of the quadrants (cross in inner ellipse), and the five ~2x2° ROIs from each region. S = superior, ST = superotemporal, T = temporal, IT = inferotemporal, I = inferior. Images were contrast-stretched for display purposes only.

The most superficial OCTA slab (50 μm deep from the inner limiting membrane [ILM]) (Fig 1B) from AngioVue was used for image analysis in order to isolate only the most superficial layer of RPCs. For each of the eyes imaged, the OCTA with the highest contrast and the least motion artifact (i.e. fewest vertical and horizontal artifactual lines) was selected as the reference image; the other nine OCTAs were registered to this reference image using the Register Virtual Stack Slices plug-in on Image J; we employed a rigid extraction model with elastic bUnwarpJ splines registration [41]. For sets of images in which registration failed using the superficial OCTA slab due to low contrast, a deeper layer with larger blood vessels (vitreous to 150 μm below the ILM) was registered, and the transformations from this set of deep layer images was then applied to the superficial OCTAs using the Transform Virtual Stack Slices plug-in on ImageJ.

### Defining regions of interest and averaging

**Fig 2:**
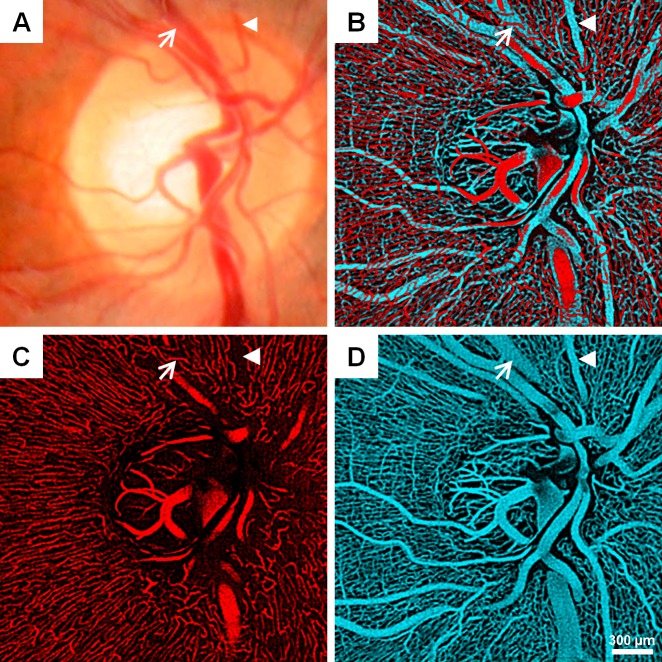
Qualitative features of the RPC network in a healthy control. (A) Fundus photograph. (B) Overlay of superficial (red; ILM to 50-μm-deep slab) and deep (cyan; ILM to 150-μm-deep slab) vasculature around the optic disc displayed separately in (C) and (D), respectively. Arterial and venous vessels (arrowhead and arrow, respectively) are identified on each image. Note what looks like an area of non-perfusion on the superficial layer actually overlies a deeper artery seen in (D) (arrowhead) and the capillaries course over a large vein in (D) (arrow).

First, the optic disc margin was manually approximated into an ellipse based on the choroidal OCTA layer (75 μm above the retinal pigment epithelium to the end of the OCT image) using a custom MATLAB program (The MathWorks Inc., Natick, MA) (Fig 1A and 1B). The regions of interest (ROIs) were defined as ~2x2° (60x60 pixel) boxes centered ~1° from the optic disc margin located in areas superior, superotemporal, temporal, inferotemporal, and inferior to the disc (Fig 1C). The ROIs were adjusted manually within these areas in order to include only capillaries and avoid major vessels. Capillary-free zones around arterial vessels may appear as artifactual areas of capillary dropout on this superficial slab if there is a deeper artery (Fig 2). Therefore, deeper OCTA layers were also checked when placing ROIs in order to avoid these zones. Within each ROI, the OCTAs were averaged to create 2- to 10-frame averaged images. If the ROI contained one or more frames of poor quality (e.g. excessive eye motion) or frames with areas missing within the ROI (usually in superior or inferior regions), the entire ROI was excluded from analysis. Of the 110 possible ROIs imaged, 11 ROIs were excluded due to poor image quality or partial coverage.

### Computation of signal-to-noise ratio

**Fig 3:**
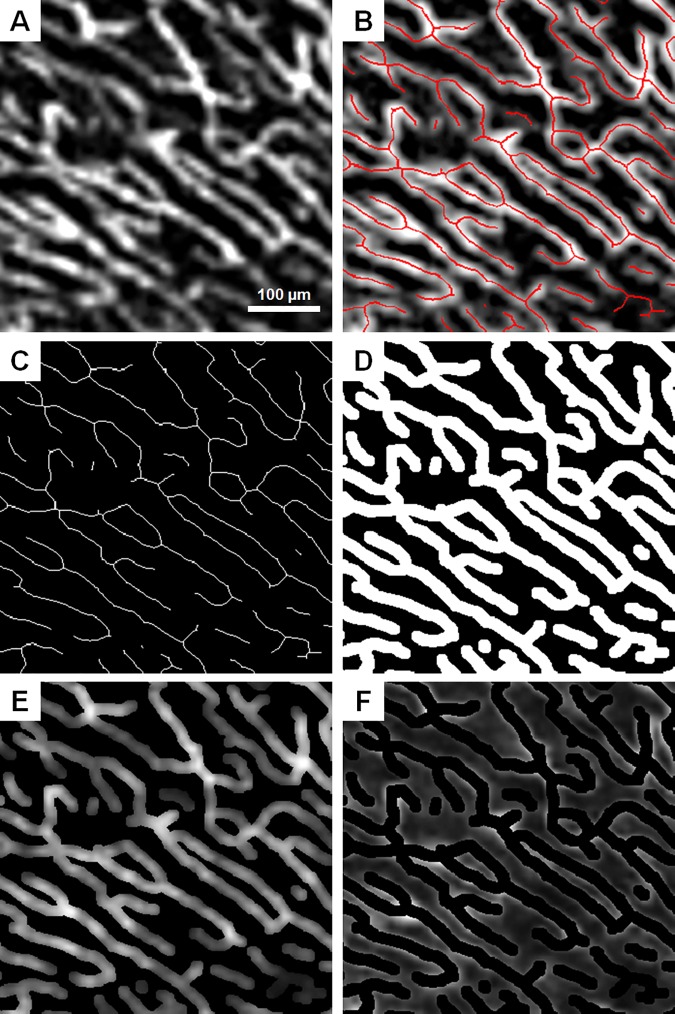
Signal and background intensity extraction using mask-based segmentation on the 10-frame averaged ROI. (A) 10-frame averaged ROI. (B) ROI with skeletonization superimposed in red. (C) Skeleton only. (D) Skeleton dilated to 15 μm in diameter. (E) Signal mask from dilated skeleton superimposed with the ROI. (F) Background mask from inverse of dilated skeleton superimposed with the ROI. Images were contrast-stretched for display purposes only.

Signal and background regions were defined on the 10-frame averaged for each ROI using a mask-based segmentation (Fig 3) [42]. First, the 10-frame averaged ROI was resized by a factor of 6 to 360x360 pixels using bicubic interpolation. The 10-frame averaged ROI was then contrast stretched, and adaptive thresholding over 50x50 pixels was performed (Fig 3A). Next, the thresholded image was skeletonized (Fig 3B and 3C). The skeleton was dilated to approximately 15 μm in diameter in order to create a mask (Fig 3D). This 15-μm diameter capillary mask was chosen based on previous histological studies that have sized RPCs at about 9 μm in diameter [2, 26] and our previous work that OCTA overestimates the diameter of vessels by approximately 6 μm [23]. Signal was defined as the region within the dilated skeleton mask, whereas background was defined as the inverse of the mask (Fig 3E and 3F). This mask generated from the 10-frame averaged ROI was applied on the +corresponding reference and each averaged ROI.

Mean and standard deviation of the pixel intensity within the signal and background regions were obtained for the single-frame and each of the averaged images. Signal-to-noise ratio (SNR) was then calculated using the following formula:

$$SNR=\frac{\mu_{I_{signal}}-\mu_{I_{background}}}{\sqrt{\sigma_{I_{signal}}^{2}+\sigma_{I_{background}}^{2}}}$$

Where μ_*I*_ is the mean intensity and *σ*_*I*_ is the standard deviation of the intensity.

### Capillary endpoints and mean capillary segment length

**Fig 4:**
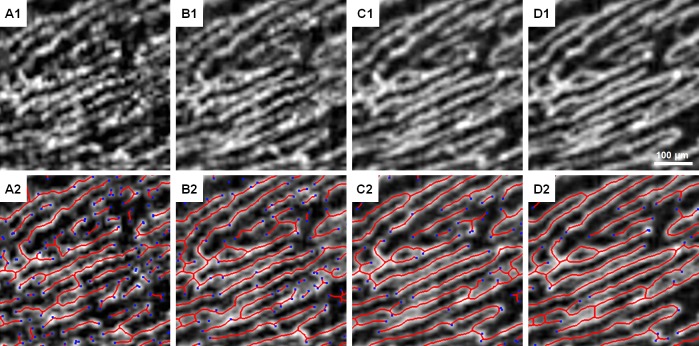
Skeletonization and endpoint identification on different numbers of averaged frames from the same ROI. (A1 & A2) Reference frame. (B1 & B2) 2-frame averaged. (C1 & C2) 5-frame averaged. (D1 & D2) 10-frame averaged. Blue dots denote endpoints of the skeleton in red. Images were contrast-stretched for display purposes only.

Skeletonizations were generated for the reference and each averaged (2- to 10-frame averaged) ROI in the same manner as the 10-frame averaged skeleton generated for the mask used to define signal and background regions. The skeletons were then used to automatically generate the number of endpoints (Fig 4) using the MATLAB function bwmorph. The mean length of segments was calculated as the total length of the skeleton divided by the number of segments with each segment defined as continuous pixels between endpoints and/or branch points generated from the bwmorph function.

### Capillary density and intercapillary distance

**Fig 5:**
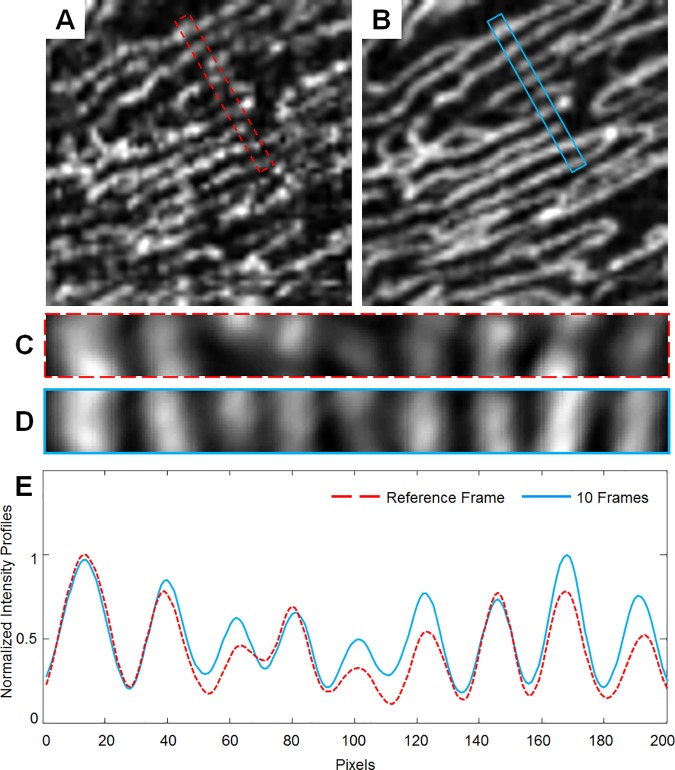
Intercapillary distance measurement. (A) Reference frame and (B) 10-frame averaged ROI with 0.1x1° sampling box outlined in red dashed and blue lines, respectively. (C & D) Sampling box from (A & B) enlarged. (E) Normalized intensity profiles generated from the sampling box. ICD is calculated as the peak-to-peak distance. Images were contrast-stretched for display purposes only.

Using the automatically generated skeletons, RPC density was calculated as the length of the pixels in the skeleton divided by the ROI area for the reference and each averaged ROI. For intercapillary distance (ICD), a 0.1x1° sampling box was placed within each ROI, positioning the longest dimension of the box perpendicular to the direction of the vessels. An averaged intensity profile was then generated from this sampling box, and ICD was defined as the mean peak-to-peak distance (Fig 5). The height of the sampling box is about twice the average diameter of capillaries. This ensures that if there were capillaries perpendicular to the RPCs, they would have reduced contribution to the averaged intensity profile and thus minimizing their impact on the peak-to-peak measurement and ICD calculation.

### Statistics

Statistical analysis was performed using SPSS 20.0 Statistical Software (IBM Corporation, Chicago, IL). One-way repeated measures analysis of variance (RM-ANOVA) with a Greenhouse-Geisser correction and post hoc testing using the Bonferroni correction were performed. Significance level was set at 0.05.

### Capillary density in glaucoma patients

Using the 10-frame averaged images, healthy control density data from each peripapillary region were used to generate a box plot, and the density data from each glaucoma patient was plotted in the corresponding regions.

## Results

### Subject characteristics

The mean ± SD age of the 22 healthy controls was 44 ± 15 years (range: 21 to 71 years). Mean ± SD age for the 3 patients with glaucoma was 60 ± 3 years.

### General RPC appearance on single-frame and averaged images

As expected, the vessels appeared smoother and more continuous with increasing number of frames. Signal that appeared to be noise on the single-frame image became clearly vessels or background after averaging (Fig 4). Motion artifacts from single-frame images became less prominent. On the averaged ROIs, the parallel organization and sparse anastomoses characteristic of the RPCs were better appreciated.

### Signal-to-noise ratio

**Fig 6:**
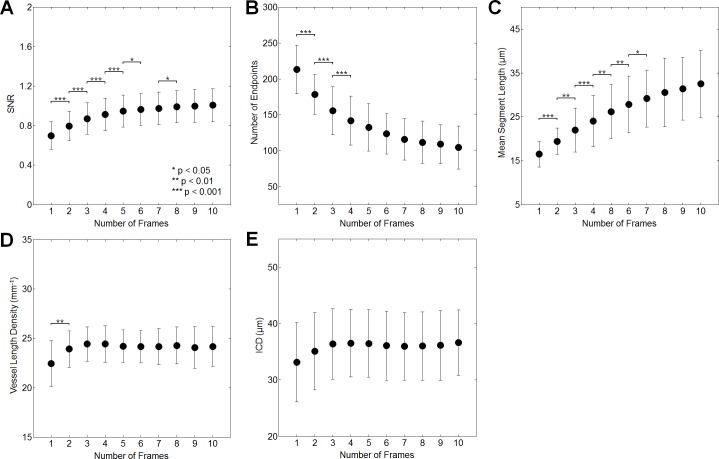
Comparison of quantitative measures among reference and averaged images in the superior region (for other regions, please see S1 Fig). Improvements in SNR (A) remain significant up to 6 frames averaged. Decreases in the number of skeleton endpoints (B) remain significant up to 4 frames averaged. Increases in the mean length of skeleton segments (C) remain significant up to 7 frames averaged. Increases in vessel density (D) remain significant up to 2 frames averaged. ICD (E) does not change significantly among frames.

When comparing the reference frame to the 10-frame averaged ROI, SNR increased by 44.1% superiorly, 46.1% superotemporally, 45.8% temporally, 42.8% inferotemporally, and 43.3% inferiorly (Table 1). This represents an average increase of 44.4% in SNR for all 5 ROIs. RM-ANOVA post hoc testing revealed that no further improvement in SNR was seen beyond averaging 6 frames superiorly, 9 frames superotemporally, 5 frames temporally, 8 frames inferotemporally, and 5 frames inferiorly (Fig 6; S1 Fig; Table 2).

**Table 1:** Percent difference from the reference to 5-frame and 10-frame averaged ROI in quantitative metrics for each region.

	Superior		Superotemporal		Temporal		Inferotemporal		Inferior	
Number of Frames	5	10	5	10	5	10	5	10	5	10
**SNR**	35.7	44.1	36.1	46.1	37.0	45.8	33.9	42.8	34.4	43.3
**Number of Endpoints**	-37.8	-50.9	-39.1	-52.2	-38.9	-51.2	-41.1	-54.0	-35.0	-47.6
**Mean Segment Length**	58.9	97.5	54.6	87.5	65.5	106.7	53.4	88.2	42.2	75.3
**Capillary Density**	7.8	7.8	10.5	10.5	13.5	13.8	12.1	13.2	8.2	7.7
**ICD**	10.0	10.5	4.1	6.5	-5.5	-4.4	1.1	1.5	8.2	8.6

SNR, signal-to-noise ratio; ICD, intercapillary distance.

**Table 2:** Optimum number of averaged frames for each quantitative metric.

	Superior	Superotemporal	Temporal	Inferotemporal	Inferior
**SNR**	6	9	5	8	5
**Number of Endpoints**	4	4	6	5	4
**Mean Segment Length**	7	6	5	5	5
**Capillary Density**	2	2	3	2	2
**ICD**	1	1	1	1	1

SNR, signal-to-noise ratio; ICD, intercapillary distance.

### Skeletonization metrics

When comparing the reference to the 10-frame averaged ROI, the number of skeleton endpoints decreased by 50.9% superiorly, 52.2% superotemporally, 51.2% temporally, 54.0% inferotemporally, and 47.6% inferiorly (Table 1). This represents an average decrease of 51.2% for all ROIs. RM-ANOVA post hoc testing revealed that no further reduction in the number of skeleton endpoints was seen beyond averaging 4 frames superiorly, 4 frames superotemporally, 6 frames temporally, 5 frames inferotemporally, and 4 frames inferiorly (Fig 6; S1 Fig; Table 2).

The mean length of the skeleton segments increased by 97.5% superiorly, 87.5% superotemporally, 106.7% temporally, 88.2% inferotemporally, and 75.3% inferiorly in comparing the reference to the 10-frame averaged ROI (Table 1). This represents an average increase of 91.0% across all ROI. RM-ANOVA post hoc testing revealed that no further increase in the mean skeleton segment length was seen beyond averaging 7 frames superiorly, 6 frames superotemporally, 5 frames temporally, 5 frames inferotemporally, and 5 frames inferiorly (Fig 6; S1 Fig; Table 2).

### Density and ICD

When comparing the reference frame to the 10-frame averaged ROI, capillary density increased by 7.8% superiorly, 10.5% superotemporally, 13.8% temporally, 13.2% inferotemporally, and 7.7% inferiorly (Table 1). This represents an average increase of 10.6% over all ROI. RM-ANOVA post hoc testing revealed that no further increase in capillary density was seen beyond averaging 2 frames superiorly, 2 frames superotemporally, 3 frames temporally, 2 frames inferotemporally, and 2 frames inferiorly (Fig 6; S1 Fig; Table 2).

When comparing the reference frame to the 10-frame averaged images, ICD changed by 10.5% superiorly, 6.5% superotemporally, -4.4% temporally, 1.5% inferotemporally, and 8.6% inferiorly (Table 1). This represents an average change of 4.5% across all ROI. Mean ICD data from the superior and superotemporal regions were significant on RM-ANOVA, but post hoc testing in these regions revealed no significant pair-wise differences in comparing sequential numbers of frames averaged. The other three regions showed no significant differences on RM-ANOVA (Fig 6; S1 Fig; Table 2).

### Capillary density in glaucomatous eyes

**Fig 7:**
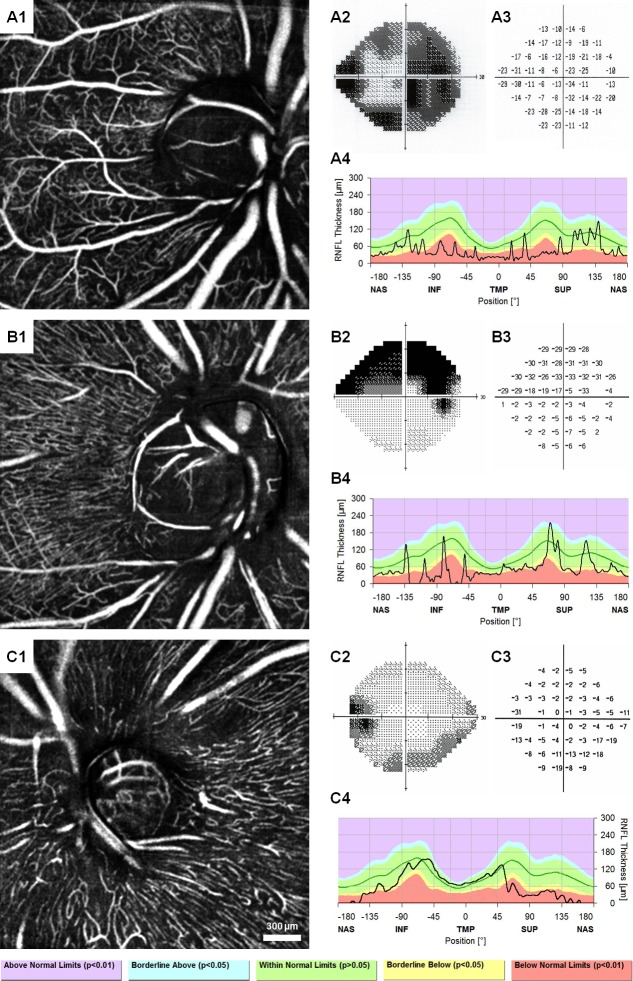
Ten-frame averaged OCTA, Humphrey visual fields with total deviation, and peripapillary RNFL thickness profiles of three patients with POAG. OCTA_0169 shows the loss of the superficial RPC layer and revealing of the deeper vascular network due to severe RNFL thinning (A1), corresponding with diffuse visual field defects (A2 & A3) and RNFL thinning (A4). OCTA_0171 shows loss of RPC layer superiorly, inferotemporally, and inferiorly on OCTA (B1), somewhat corresponding to a superior hemifield defect (B2 & B3) and prominent RNFL thinning in the inferior region with borderline values in the other regions (B4). JC_10897 shows loss of the RPC layer superficial and superotemporal to the disc (C1), corresponding to the inferior arcuate defect (C2 & C3) and RNFL thinning superiorly and superotemporally (C4). Images were contrast-stretched for display purposes only.

**Fig 8:**
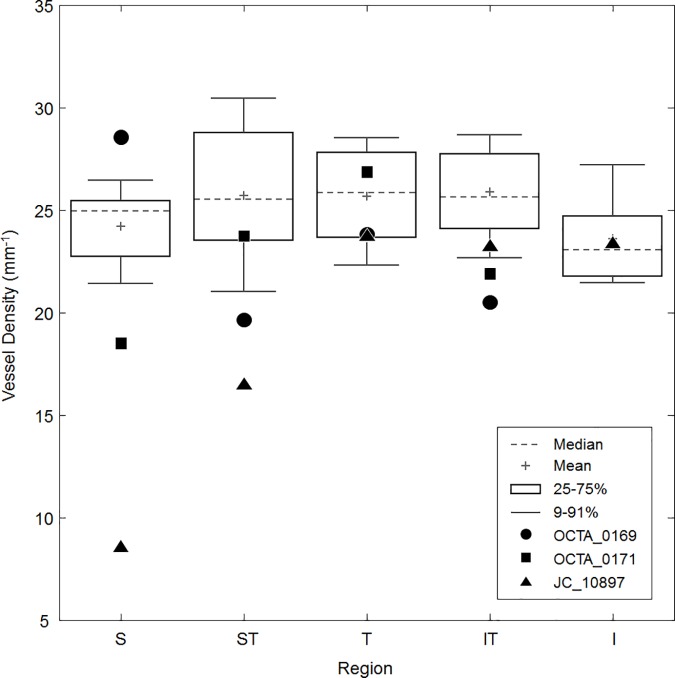
Box plot of healthy control vessel density data generated from 10-frame averaged images overlaid with data from three patients with POAG. OCTA_0169 (circle) has low density in all regions except superiorly, roughly corresponding the diffuse visual field defects and RNFL thinning from Fig 6. OCTA_0171 (square) has low density in all regions except temporally; this pattern is well-correlated with the RNFL thickness plot, though the visual field does show some minor defects in the superior and superotemporal regions. JC_10897 (triangle) shows densities well below normal in the superior and superotemporal regions, corresponding to the inferior visual field defect and superior RNFL thinning. Density was not measured in the inferior region for OCTA_0169 and OCTA_0171 due to constraints of the image size and presence of many large vessels.

Fig 7 shows the 10-frame averaged OCTA images of each subject with POAG in addition to their Humphrey visual fields and RNFL thickness plots. Compared to controls, there was lower density in regions corresponding to visual field defects and RNFL thinning in the POAG patients (Fig 8). Of note, the parallel structure of the RPCs seen in the healthy controls was replaced by a mesh-like network of vessels and there were more large caliber vessels visible in the glaucomatous eyes compared to controls.

## Discussion

In this study, we showed that OCTA image registration and averaging 1) increased SNR significantly up to 5–9 frames averaged, 2) improved skeletonization metrics up to 4–7 frames averaged, 3) increased density up to 2–3 frames averaged, and 4) had no significant effect on ICD. This work is a novel application of a well-established approach to improve image quality, and we were able to use quantitative parameters to evaluate this improvement and its impact on vascular measures such as capillary density.

Histologically, the RPCs form a two dimensional network within the superficial portion of the RNFL and have a characteristic appearance: long, straight capillaries with few anastomoses running parallel to the nerve fiber bundles [1, 2, 8, 24, 43, 44]. The vessels imaged in the superficial 50-μm slab for this study are morphologically consistent with this description, and we did not observe the net-like configuration of the deeper vessels in the healthy controls. Moreover, they course over large venous vessels but exhibit capillary-free zones around arterial vessels as previously observed (Fig 2). Using our technique, we were also able to remove some of the image artifacts, such as discontinuous vessel segments, non-uniform appearance, and eye motion artifacts. Quantitatively, we found that mean ICD of the RPCs ranged from 30 to 40 μm, which is in agreement with prior histological studies [2, 26]. Our regional ICD patterns were similar to findings from Yu *et al*. in that ICD was lowest in the superotemporal, temporal, and inferotemporal areas on OCTA [26]. Prior reports on peripapillary vessel density using histology and OCTA have utilized area coverage density in contrast to our vessel length density method. In order to compare our data from earlier findings, we can estimate area coverage density by assuming that RPC diameter is uniformly 9 μm (based on previous histologic reports) and mathematically converting vessel length density. This calculation yields mean values between 21 and 24% for our study. These values are within the 10–30% range seen on histology [2, 43, 44].

Our results suggest that the number of frames needed for averaging varies depending on the measurement of interest. For higher quality images and accurate vascular segmentation, at least 4–5 frames are necessary. These factors may be important in investigating vascular branching patterns through fractal analysis, for example, especially if automated methods are used to model the vasculature. On the other hand, density and ICD require only 1–3 frames to generate consistent data. A possible explanation for the ICD findings may be that our use of an averaged intensity profile within a 2-dimensional sampling box instead of along a line was in effect similar to averaging the image itself. Moreover, the larger the height of the sampling box, the more averaged the results would be. This effect may explain why there was no significant effect of our technique on ICD measurements. Capillary density and ICD can be used to study vascular changes in pathology involving the RPC network, such as glaucoma or ischemic retinal diseases. Our method of measuring ICD in particular has the advantage of not requiring image averaging to produce reliable data, and thus can be used on single scans as a local indication of capillary density in images with relatively poorer quality.

Furthermore, we observed differences in the number of averaged frames needed to optimize our parameters among the peripapillary regions, especially in SNR. The increased number of frames needed to optimize SNR in the ROIs with diagonally-oriented vessels (i.e. superotemporal and inferotemporal) may be due to the slight differences in mask dilation between these regions and the horizontally- or vertically-oriented regions. Since the width of the mask is rounded to the nearest single pixel, it is slightly wider in the superotemporal and inferotemporal ROIs, leading to inclusion of the edges of the vessels which have more variations in intensity than the center. This slight difference in segmentation may be the reason for the higher number of frames needed to optimize SNR. The variations in the optimization of the other parameters are relatively small. It is possible that with a larger sample size, this difference may become negligible.

The limitations to this study are as follows. 1) The mask-based segmentation with a uniform capillary mask diameter does not perfectly segment background and signal. There may be residual signal in the background and vice versa (Fig 3E and 3F). 2) The OCTA instrument used in this study currently requires that the subject have excellent fixation. This issue may be resolved with eye motion tracking. 3) Our method of setting the lower limit of the OCTA slab as 50 μm below the ILM results in the inclusion of deeper, non-RPC vessels in patients with RNFL thinning, as seen in the POAG subjects in this study (Fig 7). This phenomenon may lead to falsely normal vessel density in some patients despite obvious RPC loss and limits our ability to use ICD as a quantitative measure. 4) In peripapillary regions with thicker RNFL, such as superiorly and inferiorly, we may be missing some deeper RPCs by using the uniform 50-μm slab from the ILM. 5) The manual sampling placement for ICD may have not reflected the ICD in the whole region. A possible solution to this limitation is perhaps utilizing a sliding sampling window moving perpendicularly to the RPCs to sample all possible ICDs at the peripapillary region.

## Conclusions

Image registration and averaging is a simple method to enhance the overall appearance and quantitative outputs of RPCs from OCTA. This technique allows clinicians and researchers to use an existing instrument to produce reliable, meaningful angiographic data. Moreover, by finding minimal numbers of frames needed for various quantitative parameters, we can maximize patient comfort without compromising the quality of our data. With improvements in eye motion tracking and signal detection, perhaps even fewer frames will be necessary. Using this technique, future studies will be able to extract more reliable data from OCTA in order to elucidate the role of peripapillary microvascular changes in diseases such as glaucoma.

## Supporting Information

S1 Fig Comparison of quantitative measures among reference and averaged images in the superotemporal (ST), temporal (T), inferotemporal (IT), and inferior (I) regions. Improvements in SNR (1^st^ column) remain significant up to 5 to 9 frames averaged. Decreases in the number of skeleton endpoints (2^nd^ column) remain significant up to 4 to 6 frames averaged. Increases in the mean length of skeleton segments (3^rd^ column) remain significant up to 5 to 6 frames averaged. Increases in vessel density (4^th^ column) remain significant up to 2–3 frames averaged. ICD (5^th^ column) does not change significantly among frames.
(TIF)

S1 Table Data from each quantitative measure used for analysis. Each spreadsheet A-E contains data from its corresponding parameter for each subject, ROI, and number of averaged frames. Spreadsheet F contains the 10-frame averaged capillary density data for the patients with POAG. Mean segment length and intercapillary distance (ICD) are measured in μm; capillary density is measured in mm^-1^.
(XLSX)
