# Supplementary figures and images for: Visualization of Radial Peripapillary Capillaries Using Optical Coherence Tomography Angiography: The Effect of Image Averaging
Source: PLoS One. 2017 Jan 9;12(1):e0169385. doi: 10.1371/journal.pone.0169385 (PMC5222511; doi:10.1371/journal.pone.0169385)

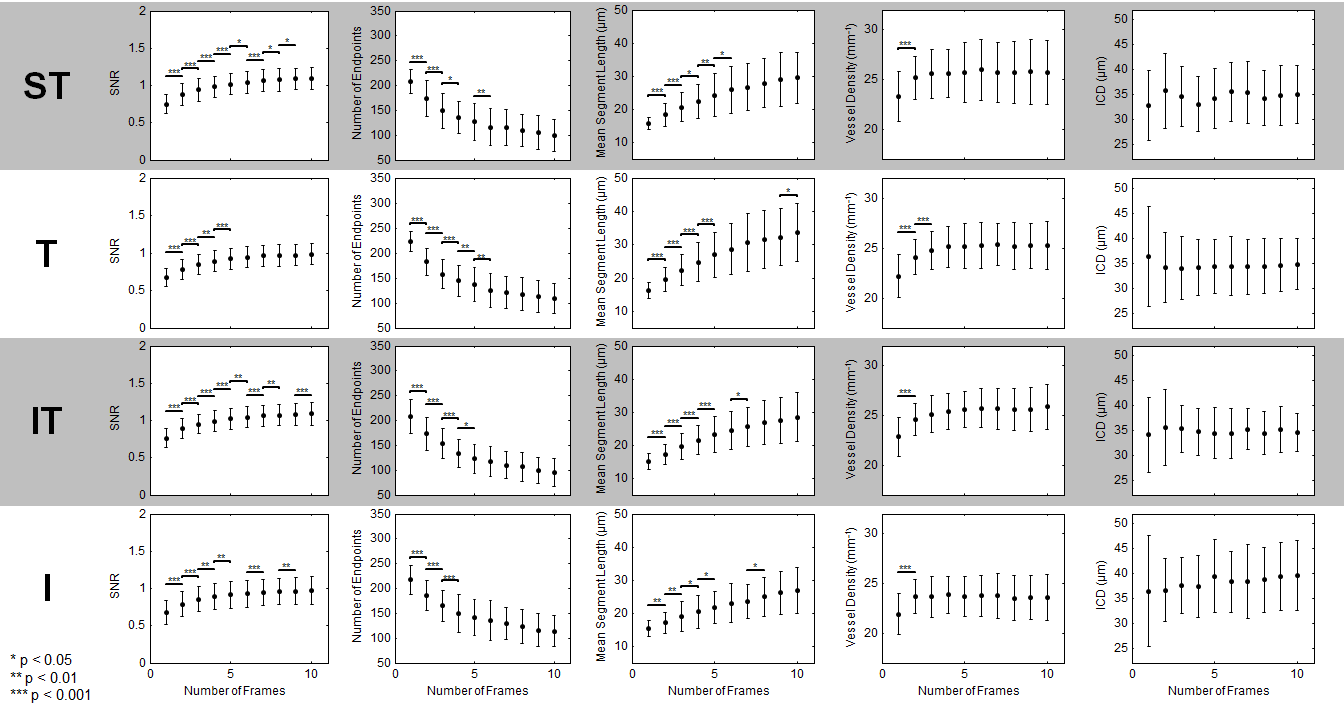

Supplement: S1 Fig — Improvements in SNR (1st column) remain significant up to 5 to 9 frames averaged. Decreases in the number of skeleton endpoints (2nd column) remain significant up to 4 to 6 frames averaged. Increases in the mean length of skeleton segments (3rd column) remain significant up to 5 to 6 frames averaged. Increases in vessel density (4th column) remain significant up to 2–3 frames averaged. ICD (5th column) does not change significantly among frames. (TIF) [file pone.0169385.s001.tif]
